# Single-shot measurement of longitudinal phase space beam profile in an electron storage ring

**DOI:** 10.1107/S1600577521011127

**Published:** 2022-01-01

**Authors:** J. Kim, G. S. Jang, B. H. Oh, J. Lee, S. Shin

**Affiliations:** a SLAC National Accelerator Laboratory, Menlo Park, CA 94025, USA; b Department of Physics, POSTECH, Pohang, Gyungbuk 37673, Republic of Korea; c Pohang Accelerator Laboratory, POSTECH, Pohang, Gyungbuk 37673, Republic of Korea

**Keywords:** longitudinal emittance, phase space profile, storage ring

## Abstract

A novel scheme to measure the longitudinal emittance and phase space profile in an electron storage ring by using correlations between time and the vertical coordinate, and between energy and the horizontal coordinate, is proposed. This longitudinal profile measurement scheme will help to demonstrate recent results of advanced studies for manipulating the longitudinal beam profile and for investigating beam instability in an electron storage ring.

## Abstract 2

A novel scheme to measure the longitudinal emittance and phase space profile in an electron storage ring by using correlations between time and the vertical coordinate, and between energy and the horizontal coordinate, is proposed.

## 1. Introduction

Transverse bunch crabbing using a two-frequency crab cavity scheme (Zholents, 2015 ▸; Huang *et al.*, 2019 ▸) provides the optimal solution to produce short-pulse (1–10 ps full width at half-maximum) X-rays in a storage ring. This scheme would enable increased precision of timing-mode studies of a large number of dynamic processes in materials as they function. A crab cavity can also be used as an injection kicker in a new on-axis injection scheme (Kim *et al.*, 2019 ▸) that uses a transverse deflecting radio-frequency (RF) cavity to kick the incoming beam into an already populated bucket but with a timing offset from the synchronous phase.

A crab cavity can also be used to measure bunch length (Loew & Altenmueller, 1965 ▸; Emma *et al.*, 2000 ▸). A crab cavity couples the *y–z* or *x–z* planes, so bunch length is projected in the *y* or *x* dimension, respectively. The resolution of this bunch length measurement is limited by transverse emittances ɛ_*x*_ and ɛ_*y*_ unless a specialized beam transport line such as a chicane (Xiang & Ding, 2010 ▸) is used. Regardless of using a crab cavity, the distribution of the energy deviation 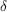 = 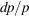 of a bunch can be projected in the *x* dimension at which horizontal dispersion η_*x*_ is large. The resolution of this is limited by the ratio ɛ_*x*_/η_*x*_. Combining these two principles enables measurement of the longitudinal profiles (*z*–δ) of a bunch in the *x*–*y* plane.

A fourth-generation storage ring (4GSR) is an accelerator that provides small emittance so that the measurement can be made with high resolution. A 4GSR adopts a multi-bend achromat (MBA), that effectively suppresses natural emittance. The MAX IV 4GSR is currently operating; others, including APS-U, SPring-8-II, SLS-II, ALS-U, SIRIUS, ESRF-EBS and Korea-4GSR, are being designed, constructed or commissioned [Streun, 2017 ▸; Steier *et al.*, 2016 ▸; Liu *et al.*, 2013 ▸; see also design reports for MAX IV (https://www.maxiv.lu.se/acceleratorsbeamlines/accelerators/acceleratordocumentation/max-iv-ddr), APS Upgrade (https://www.aps.anl.gov/APSUpgrade), SPring-8-II (http://rsc.riken.jp/eng/index.html) and ESRF-EBS (https://indico.psi.ch/event/5589/)]. All of these rings use the MBA concept. A single cell of an MBA lattice has M-1 dispersion maxima with similar amplitudes, and one of these maxima can be used as a watching point for the measurement using a crab cavity. A hybrid MBA lattice such as APS-U, ESRF-EBS or Korea-4GSR provides two large dispersion bumps at the edge of a cell to enable effective correction of chromaticity, and the region of the large dispersion bumps is a desirable choice as a watching point.

In this study, we show that in a storage ring that uses a hybrid MBA lattice the measurement on longitudinal bunch profiles gives good resolution without the need for other additional magnets. In Section 2, we recall the matrix formalism between two arbitrary points of a storage ring, and projection of longitudinal (*z*–δ) beam profiles on the transverse (*x*–*y*) plane by using a crab cavity. In Section 3, we briefly introduce PAL-4GSR, which is used as an example lattice for the novel measurement scheme. We also calculated the intrinsic resolution of the measurement in PAL-4GSR. In Section 4, we present a numerical simulation of the measurement of the longitudinal profile by using the PAL-4GSR lattice. We also examine the resolution of the measurement when wakefield data are included.

## 2. Coupling of the *y*–*z* plane

Projection of the *z*–δ beam profile into the *x–y* plane can be described by linear optics theory. A thin crab cavity of TM110 mode has a linearized function as follows (Huang, 2016 ▸),

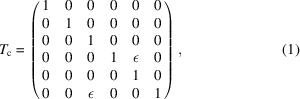

where ε = 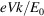 , in which *e* is the electron charge, *V* [V] is the maximum voltage of a crab cavity, *k* is the angular wavenumber and *E*_0_ [eV] is the nominal energy of a beam. In a storage ring, a linear matrix from arbitrary position 1 to arbitrary position 2 is given as (Chao, 2002 ▸)

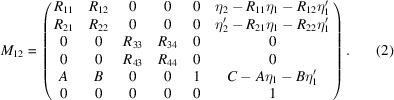

Definitions of each matrix element can be found in Appendix *A* [App appa]. When initial beam coordinates at the position 1 are given as *X*_1_ = (*x*_1_, 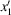 , *y*_1_, 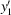 , *z*_1_, δ_1_), its continuous mapping via *T*_c_ and *M*_12_ becomes

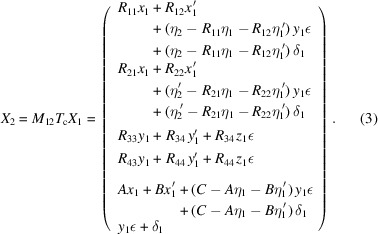

Let us assume that position 1 is located at one of the achromats of a storage ring (η_1_ = 0), and position 2 is at a point that has large dispersion. If ψ_*x*_ ≃ (2*n* + 1)π and ψ_*y*_ ≃ 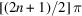 , contributions from *R*_12_ and *R*_33_ are negligible. The magnitude of *R*_11_ is approximately 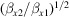 when ψ_*x*_ ≃ (2*n* + 1)π, and it is conservatively less than 3 as the ratio of β_*x*2_ over β_*x*1_ is less than 10 for most lattices. Specifically, when emittance ≃ 100 pm, δ_1_ ≃ 0.001 and η_2_ ≃ 0.10 m, the contribution from 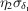 at *x*_2_ is one order larger than that from 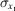 (*i.e.* 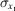 ≃ 10^−5^ m and 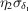 ≃ 10^−4^), which means we can expect projection between *x*_2_ and 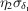 . Likewise, *y*_2_ is dominated by *R*_34_ *z*_1_ε as 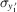 is of the order of 10^−6^ but *z*_1_ is of the order of 10^−3^ and ε has order 10^−2^. Clearly, smaller emittance will lead to better resolution.

Hence, *x* and *y* coordinates at position 2 are expressed as

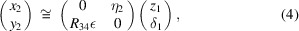

which shows the projection of the *z*–δ plane onto the *x–y* plane. Its resolution is mainly dependent on whether the contributions from *x*_1_, 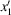 , *y*_1_ and 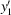 are negligible and whether ψ_*x*_ ≃ (2*n* + 1)π and ψ_*y*_ ≃ 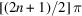 . If they are, then *R*_34_ has sin(ψ_*y*_) dependence, so large η_2_ leads to increased magnification for the *x* plane, and ψ_*y*_ close to 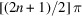 leads to increased magnification for the *y* plane.

A hybrid MBA lattice such as ESRF-EBS, APS-U, PAL-4GSR satisfies the above-described conditions. Due to the common (ψ_*x*_, ψ_*y*_) = (3π, π) phase advance between two dispersion bumps in a cell of a hybrid MBA lattice, each cell has a similar phase advance. Specifically, a hybrid MBA lattice has φ_*x*_ ≃ 0.9π and φ_*y*_ ≃ 0.4π from the center of the long straight section to the nearest dispersion bump. Hence, choosing the position of a crab cavity satisfying φ_*y*_ = 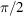 is accompanied by φ_*x*_ ≃ π.

## 3. PAL-4GSR and the intrinsic resolution

**Figure 1:**
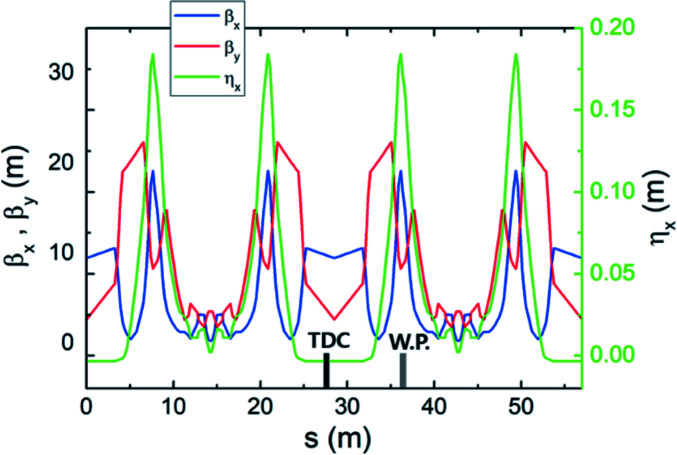
Twiss functions of PAL-4GSR (two cells) and the position of the transverse deflecting cavity and watching point.

The PAL-4GSR storage ring is a hybrid seven-bend achromat (H7BA) lattice with a horizontal emittance of 90 pm. The ring has a circumference of 570 m, and is composed of 20 symmetrical cells. From experience on PLS-II (Shin *et al.*, 2013 ▸), the length of the straight section is considered to be 6.5 m to accommodate two SCRF modules in one straight section. The PAL-4GSR lattice (Table 1 ▸, Fig. 1 ▸) contains a 2 T super-bend in the central dipole to produce radiation with a critical energy of 12 keV.

The concepts of the ESRF-EBS and APS-U lattices were adopted in the PAL-4GSR lattice. The dispersion was deliberately enlarged between the first and second dipoles and between the sixth and seventh dipoles, then three chromatic sextupoles were located in this dispersion bump region to reduce the strength required to control the chromaticity. The betatron phase advances between the two dispersion bumps were set to Δφ_*x*_ ≃ 3π in the horizontal plane and Δφ_*y*_ ≃ π in the vertical plane; as a result, nonchromatic effects of the sextupoles are canceled out naturally. To minimize natural emittance, five-step longitudinal gradient dipoles and reverse bending magnets were considered (Streun & Wrulich, 2015 ▸; Nagaoka & Wrulich, 2007 ▸; Delahaye & Potier, 1989 ▸; Streun, 2014 ▸).

**Figure 2:**
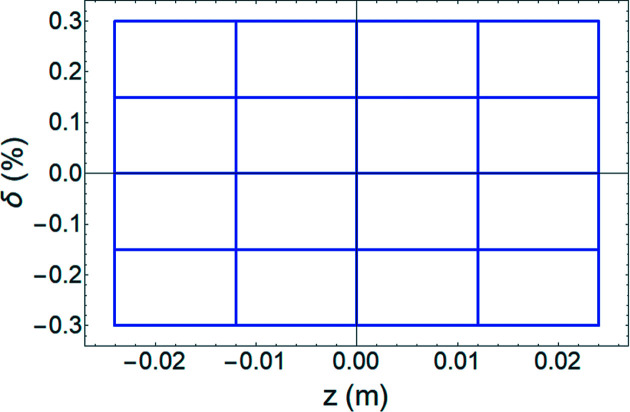
Longitudinal profiles of 100000 particles at the position of the crab cavity for examination of intrinsic resolution. Particles are distributed with a delta function in longitudinal phase space.

**Figure 3:**
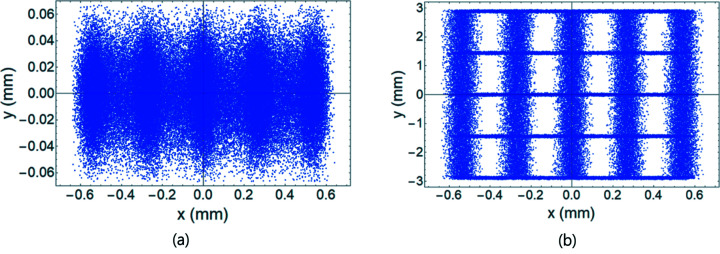
Beam distribution at the watching point with 100000 particles prepared for examination of intrinsic resolution. (*a*) TDC off; (*b*) TDC on.

**Figure 4:**
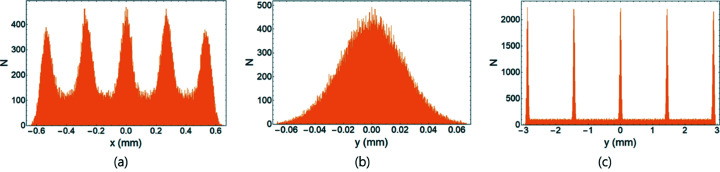
Density distribution on the *x* and *y* axis for the beam distribution shown in Fig. 3 ▸. (*a*) Density on the *x* axis when the TDC is on or off, (*b*) density on the *y* axis when the TDC is off, and (*c*) density on the *y* axis when the TDC is on.

We performed tracking simulation to examine the resolution of the measurement using a transverse deflecting cavity (TDC) on PAL-4GSR. For tracking simulation, we used *elegant* software (Borland, 2000 ▸). A total of 100000 particles were generated at the position of the TDC (Fig. 1 ▸). They have a 58 pm (standard deviation) Gaussian distribution with matched Twiss functions in the horizontal and vertical planes, and ten lines of uniform density with maximum values of ±0.3% and ±24 mm in the longitudinal plane (Fig. 2 ▸). A watching point located at the left dispersion bump has a vertical phase advance of 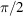 from the position of the TDC. For a TDC, we set a voltage of 4.5 MV and a frequency of 750 MHz, which yield ε = 0.02358. When the TDC was off, we observed five resolved distributions on the *x* axis at the watching point due to the correlation *x*_2_ ≃ η_2_δ_1_ [Figs. 3 ▸(*a*) and 4 ▸(*a*)]. In the *y* axis, the distribution was not resolved [Figs. 3 ▸(*a*) and 4 ▸(*b*)]. Standard deviations of Gaussian peaks were 0.037 mm on the *x* axis and 0.022 mm on the *y* axis, which are explained by 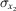 ≃ 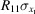 + 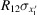 and 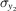 ≃ 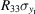 + 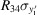 . When the TDC was on, the *x* axis was not affected, but five resolved distributions appeared on the *y* axis [Fig. 3 ▸(*b*)]. The effective length on the *y* axis increased from 0.13 mm to 5.95 mm due to *y*_2_ ≃ *R*_34_ε*z*_1_ whereas the standard deviations of the Gaussian peaks did not change. These processes allow clear resolution of the five Gaussian peaks [Fig. 4 ▸(*c*)]. From the revealed Gaussian peaks with the use of a line distribution on the longitudinal plane, we define the intrinsic resolution on an axis as *R*_I_ = (distance between adjacent Gaussian peak) / (standard deviation of a Gaussian peak). *R*_I_ increases with increase in the clarity of distinction of the Gaussian peak on an axis. PAL-4GSR has *R*_I_ = 6.69 on the *x* axis and *R*_I_ = 64.68 on the *y* axis. The dispersion bump (for δ–*x* correlation) increases and the voltage of the TDC increases (for *z–y* correlation), so the resolution can be increased. Adjustment of dispersion bumps is constrained by lattice requirements, but the voltage of the TDC can be increased further without affecting lattice requirements and we can expect much better resolution than is currently achieved.

## 4. Application on PAL-4GSR

A Gaussian bunch of 100000 particles in an equilibrium state was generated for the six-dimensional phase space with the round-beam mode. It had 58 pm emittance in both horizontal and vertical phase spaces. It should be mentioned that, for simplicity, distributed coupling errors are considered along the ring except for the region from the TDC and watching point to generate round beam. With a main RF voltage of 2.15 MV, its longitudinal distribution had σ_δ_ = 0.00108 and σ_*z*_ = 7.677 mm (Table 2 ▸). For a TDC, a voltage of 4.5 MV and a frequency of 750 MHz were also used.

### 4.1. Longitudinal emittance measurement

The longitudinal emittance can be written with the standard deviation as 

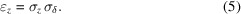

**Figure 5:**
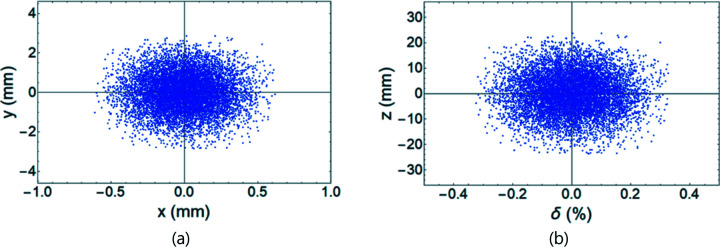
(*a*) *x–y* projection at the watching point and (*b*) longitudinal profile at the position of the crab cavity. No wakefield included.

**Figure 6:**
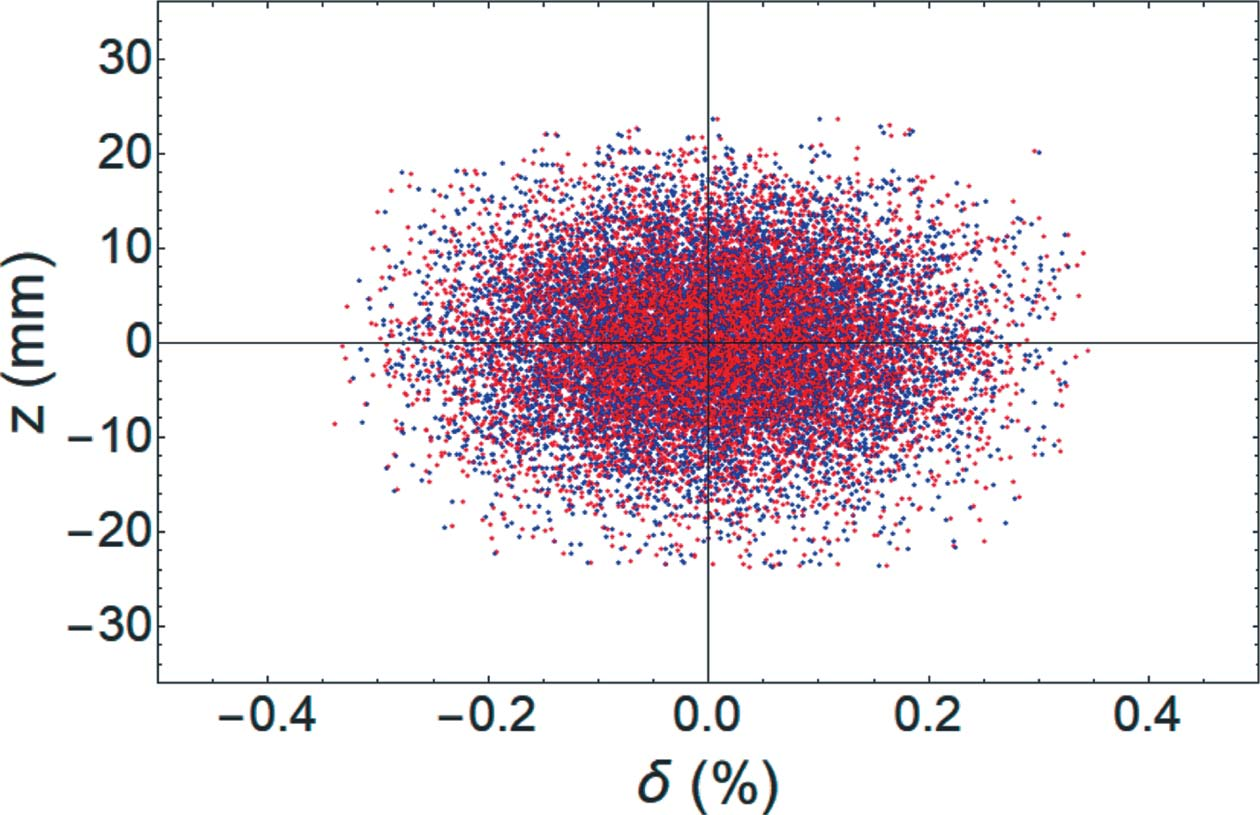
Comparison of the original longitudinal profile at the crab cavity position (blue dots) and re-projection from equation (5) (red dots). No wakefield included.

Here, using equation (4), the standard deviations σ_*z*_ and 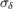 can be approximately converted to

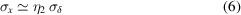

and 

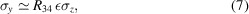

respectively. Therefore, measurements of σ_*x*_ and σ_*y*_ directly lead to estimates of longitudinal emittance. The generated Gaussian bunch is deflected at the TDC. After a 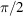 phase advance (at a watching point), the bunch’s projection on the *x–y* plane has a clear Gaussian distribution [Fig. 5 ▸(*b*)], with σ_*x*_ = 0.197 mm and σ_*y*_ = 0.922 mm. From these values, re-projection using equation (4) gives σ_δ_ = 1.096 × 10^−3^ and σ_*z*_ = 7.680 mm, which have 1.46% and 0.04%, respectively, error relative to the original values. As a result, the longitudinal emittance from the measurement is calculated to be ɛ_*z*_ = 8.417 × 10^−6^ m, which is a 1.52% error relative to the original value. The original longitudinal profile at the TDC and the profile (Fig. 6 ▸) were obtained from re-projection using equation (4).

### 4.2. Measurement of the longitudinal phase space profile with instability

**Figure 7:**
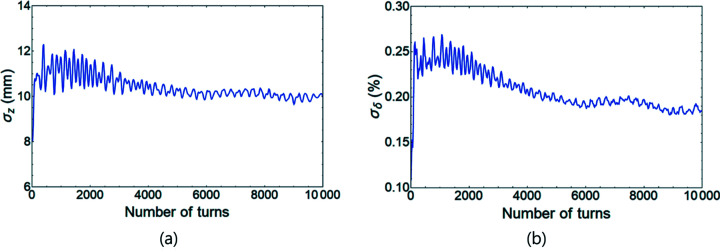
Oscillation of (*a*) r.m.s. bunch length and (*b*) r.m.s. energy spread versus turns when impedance data are included.

Beam manipulation in the longitudinal phase can be used to generate coherent radiation (Ratner & Chao, 2010 ▸) and beam dynamics in the longitudinal phase space to enable exploration of a new injection scheme (Kim *et al.*, 2019 ▸; Aiba *et al.*, 2015 ▸; Jiang *et al.*, 2016 ▸; Jiang & Xu, 2018 ▸; Kuske *et al.*, 2020 ▸). These goals invoke a need to measure the longitudinal beam profile. To generate a special shape in the longitudinal phase space, instabilities were deliberately induced by using wakefield data – here the impedance data of APS (Chae, 2003 ▸, 2007 ▸; Lindberg & Blednykh, 2015 ▸). However, PAL-4GSR and APS have different characteristics such as circumference (PAL-4GSR: 570 m; APS: 1100 m), nominal energy (PAL-4GSR: 3 GeV; APS: 7 GeV), so use of these data can invoke instabilities in PAL-4GSR. We first estimated the current threshold *I*_threshold_ of the strong instability by following Boussard’s criterion (Boussard, 1975 ▸),

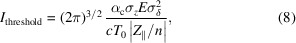

where α_c_ is the momentum compaction, σ_*z*_ is the equilibrium bunch length, 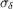 is the equilibrium relative energy spread, *c* is the speed of light, *T*_0_ is the revolution time, and 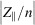 is the effective longitudinal impedance. This conservative estimate gives *I*_threshold_ ≃ 0.37 mA; therefore we used a single bunch current of 7.7 mA (or single bunch charge of 15 nC) which is sufficiently high above the estimated threshold. Including the impedance data, a tracking simulation was conducted using a Gaussian bunch of 100000 particles in the equilibrium state. Oscillations of r.m.s. bunch length and r.m.s. energy spread along a number of turns show that both quantities increased rapidly after tracking started, reached their maximum within 2000 ▸ turns and eventually converged to a new equilibrium at ∼10000 turns (Fig. 7 ▸). The new equilibrium with the impedance data has 1.3 times larger bunch length and 1.8 times larger energy spread, compared with the original equilibrium. Those results imply that strong instability was driven well.

**Figure 8:**
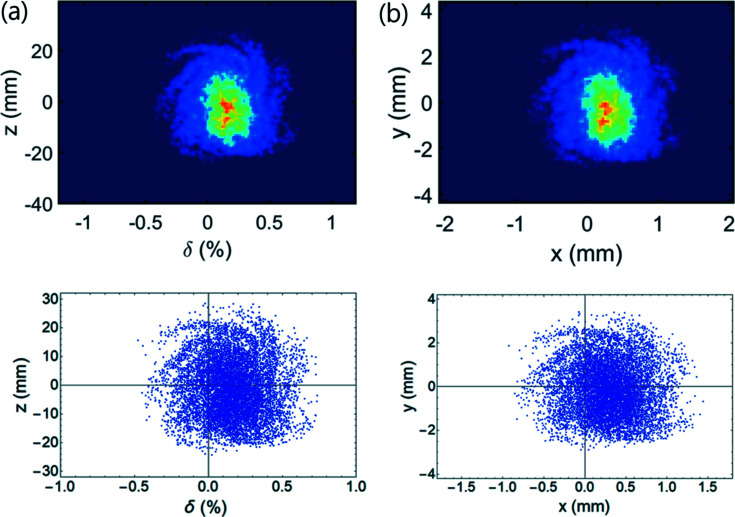
Longitudinal profiles at (*a*) the position of the crab cavity, and (*b*) the *x–y* projection at the watching point. Wakefield is included.

**Figure 9:**
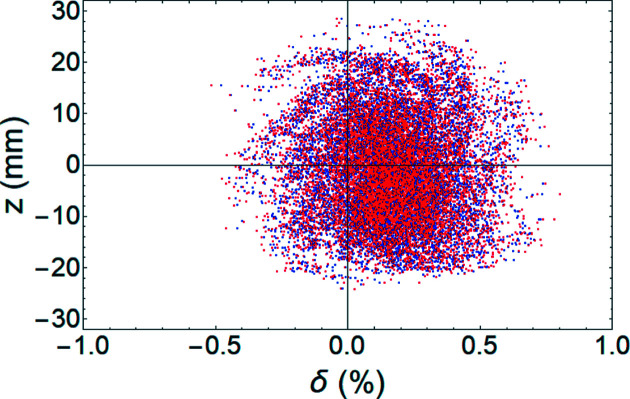
Comparison of the original longitudinal profile at the crab cavity position (blue dots) and re-projection of the *x–y* profile at the monitor position using equation (4) (red dots). Wakefield is included for a single bunch of 15 nC.

We next examined longitudinal beam profiles at 10000 turns (Fig. 8 ▸). After the kick of the TDC was applied, the longitudinal phase space was projected to the *x–y* plane. The central beam distribution split up and the outer beam distribution seems to have a spiral shape. Also, the center of the bunch was moved to ∼0.3 mm on the *x* axis. The overlap of the original longitudinal profile [Fig. 8 ▸(*a*)] and re-projection from the *x–y* plane [Fig. 8 ▸(*b*)] using equation (4) agreed well (Fig. 9 ▸).

## 5. Conclusion

We have described a novel scheme to measure the emittance and phase space profile in the longitudinal plane by using correlations between time and the vertical coordinate, and between energy and the horizontal coordinate. A large dispersion bump has a strong correlation with energy and the horizontal coordinate, and the crab cavity has a strong correlation with time and the vertical coordinate. As a result, longitudinal emittance was estimated with <1.52% error in the PAL-4GSR lattice and micro-bunching instability was observed at the synchrotron radiation source point. This longitudinal profile measurement scheme will help to guide the manipulation of beams in longitudinal phase space.

## Figures and Tables

**Table 1:** Parameters relevant to the PAL-4GSR lattice

Parameter	Value	Unit
Energy	3	GeV
Emittance (flat / round)	90 / 58	pm
Circumference	569.96	m
Tune (*x* / *y*)	47.545 / 18.203	–
Natural chromaticity (*x* / *y*)	−96.0 / −57.9	–
Radiation loss per turn	0.468 (without insertion device)	MeV
Momentum compaction	1.45 × 10^−4^	
Damping partition (H / V / L)	1.82 / 1.0 / 1.18	
Damping time (H / V / L)	13.37 / 24.35 / 20.66	ms
Main RF voltage	2.15	MV

**Table 2:** Particle distribution at the equilibrium

Parameter	Value	Unit
Horizontal emittance	58	pm
Vertical emittance	58	pm
Energy spread (r.m.s.)	0.00108	–
Bunch length (r.m.s.)	7.677	mm

## Appendix A Definitions of each matrix element of equation (2)

α, β are typical beta functions, η, η′ are dispersion functions, with corresponding subscript. ψ_*x*_ and ψ_*y*_ are, respectively, the horizontal and vertical phase advances between positions 1 and 2.
